# Sensitivity of Mouse Lung Nuclear Receptors to Electronic Cigarette Aerosols and Influence of Sex Differences: A Pilot Study

**DOI:** 10.3390/ijerph21060810

**Published:** 2024-06-20

**Authors:** Shikha Sharma, Dustin Rousselle, Erik Parker, Carolyn Damilola Ekpruke, Rachel Alford, Maksat Babayev, Sarah Commodore, Patricia Silveyra

**Affiliations:** 1 Department of Environmental and Occupational Health, School of Public Health, Indiana University, Bloomington, IN 47405, USA; shikshar@iu.edu (S.S.); drousse@iu.edu (D.R.); rachalfo@iu.edu (R.A.); mbabayev@iu.edu (M.B.); scommod@iu.edu (S.C.); 2 Biostatistics Consulting Center, Department of Epidemiology and Biostatistics, School of Public Health, Indiana University, Bloomington, IN 47405, USA; erikpark@indiana.edu; 3 Department of Medicine, Indiana University School of Medicine, Indianapolis, IN 46202, USA

**Keywords:** electronic cigarette, E-liquids, nuclear receptors, nicotine, sex differences, lungs, chemicals of concern in ENDS (Electronic Nicotine Delivery Systems)

## Abstract

The emerging concern about chemicals in electronic cigarettes, even those without nicotine, demands the development of advanced criteria for their exposure and risk assessment. This study aims to highlight the sensitivity of lung nuclear receptors (NRs) to electronic cigarette e-liquids, independent of nicotine presence, and the influence of the sex variable on these effects. Adult male and female C57BL/6J mice were exposed to electronic cigarettes with 0%, 3%, and 6% nicotine daily (70 mL, 3.3 s, 1 puff per min/30 min) for 14 days, using the inExpose full body chamber (SCIREQ). Following exposure, lung tissues were harvested, and RNA extracted. The expression of 84 NRs was determined using the RT^2^ profiler mRNA array (Qiagen). Results exhibit a high sensitivity to e-liquid exposure irrespective of the presence of nicotine, with differential expression of NRs, including one (females) and twenty-four (males) in 0% nicotine groups compared to non-exposed control mice. However, nicotine-dependent results were also significant with seven NRs (females), fifty-three NRs (males) in 3% and twenty-three NRs (female) twenty-nine NRs (male) in 6% nicotine groups, compared to 0% nicotine mice. Sex-specific changes were significant, but sex-related differences were not observed. The study provides a strong rationale for further investigation.

## 1. Introduction

The promotion of electronic nicotine delivery systems (ENDS), or electronic cigarettes, as “safer” alternatives to combustible tobacco products is driven in part by lower observed toxic levels in electronic cigarettes aerosols compared to cigarette smoke [1]. This has contributed to a notable shift in the United States, particularly among teenagers and young adults, from traditional tobacco cigarettes to various forms of electronic cigarettes [2,3]. While some of these trends have followed sex-specific patterns [4], the biological and toxicological effects of the various chemicals contained in ENDS in the male and female lung remains poorly understood [5].

Electronic cigarettes operate with a rechargeable lithium polymer battery, heating e-liquids containing nicotine and non-nicotine flavors to transform it into vapor, allowing users to inhale the vapor in a manner reminiscent of smoking traditional cigarettes. Mainstream electronic cigarettes commonly feature pre-filled and refillable pods with nicotine and without nicotine, providing a varied selection of e-liquid formulations with various flavors [6]. The nebulizer elements within electronic cigarettes commonly consist of coil and wick materials, often incorporating metals such as copper, silver, zinc, tin, nickel–chromium alloy, or chrome–aluminum alloy.

Due to their intricate design involving plastics, glass, and e-liquids, ENDS have the potential to include various emerging chemicals of concern (ECCs), such as degraded or dissociated nicotine in e-liquids, as well as endocrine-disrupting chemicals (EDCs), which affect the endocrine and non-endocrine mechanisms by interacting with nuclear receptors (NRs) [5,7]. Some of the ECCs found in ENDS include phthalates, heavy metals, phenolic compounds, flame retardants, nicotine, carbonyls, reactive oxygen species (ROS), tobacco-specific nitrosamines (TSNAs), aldehydes, polycyclic aromatic hydrocarbons (PAHs), minor tobacco alkaloids, flavoring chemicals, vegetable glycerin (VG), and propylene glycol (PG) [6,7,8,9,10,11]. While PG and VG are deemed ‘generally recognized as safe’ (GRAS) by the FDA for food additives, their safety is established in the context of consumption. However, when subjected to alternative routes of administration, particularly inhalation, additional risks arise [12,13,14]. For example, recent research has shown that non-phthalate plasticizers can induce endocrine-disrupting effects by engaging mechanisms regulated by NRs [15]. For these reasons, significant emphasis in current ENDS research has been directed towards scrutinizing the constituents of e-liquids, with a notable gap in the investigation of substances potentially leaching directly from the ENDS devices to the e-liquids, and the specific effects in the male vs. female lung [4]. In addition, sex-related differences in lung toxicity and diseases have been thoroughly studied and reported across the life span [16], but research on sex-specific mechanisms of ENDS effects is limited despite reports of sex and gender influences on ENDS toxicity and use [4].

Despite the prevailing perception of ENDS safety, the detection of EDCs like phthalates and heavy metals prompts questions into their safety profile [17]. Research indicates that exposure to tobacco smoking is linked to epigenetic alterations in the respiratory tract [18], as well as to respiratory conditions such as wheezing, asthma, chronic obstructive pulmonary disease (COPD), and lung cancer, mirroring the health effects associated with cigarette smoking [19,20]. Similarly, recent studies have demonstrated that exposure to e-liquids with tobacco and menthol flavors trigger inflammation and disrupt repair processes in the lung epithelium [21,22], and induce the expression of carcinogenic genes in a similar fashion than conventional tobacco smoke, even in the absence of nicotine [10,23].

NRs, including estrogen receptors (ERs), androgen receptors (ARs), progesterone receptors, thyroid receptors (TRs), retinoid receptors, and others such as orphan receptors, serve as potential targets for EDCs [24]. NRs modulate gene transcription through ligand-induced structural changes, recruiting co-activators [25]. Their conserved DNA-binding domain (DBD) and variable ligand-binding domain (LBD) mediate ligand interactions and transactivation functions. Previous research has investigated the modulation of gene expression by chemicals in e-liquids, irrespective of the presence or absence of nicotine [21,26]. NRs are the prime targets for man-made compounds that mimic or antagonize the action of endogenous ligands. They also occupy a prominent role in the OECD (set of internationally accepted guidelines for chemical testing by the Organization of Economic Co-operation and Development) [27] and US Environmental Protection Agency (EPA) guidelines [28] for testing and assessment of unregulated and regulated chemicals of concern including EDCs [29]. Therefore, we hypothesized that the harmful constituents of e-liquids and the contaminants leaching from the construction materials of ENDS enter the respiratory system through inhalation of aerosols from e-liquid/vaping juices and activate NRs, potentially altering NR-mediated endocrine and non-endocrine mechanisms in the lung. We aimed to evaluate the efficiency of detecting NRs’ expression to be used as a screening method for risk and exposure assessment of harmful chemicals present in electronic cigarettes. To test our hypothesis, we checked the expression of NRs transcripts to assess the risks associated with exposure to e-liquids and their potential to induce lung toxicity in the male and female lung. Our focus was directed to understand the effects of electronic cigarette exposure on the modulation of NRs in the lungs, in the presence or absence of nicotine, and considering sex as a biological variable. Our study underscores the importance of NR-mediated mechanisms when evaluating the risks linked to electronic cigarette exposure, potentially associated with the harmful substances found in electronic cigarettes, including degraded/dissociated nicotine in e-liquids and contaminants from the construction materials of the e-cigarettes such as endocrine-disrupting chemicals (EDCs) like phthalates, heavy metals, and flame retardants which target NRs in the male and female lung.

## 2. Materials and Methods

### 2.1. Animals

Adult male and female C57BL/6J mice (6–8 week of age) were purchased from The Jackson Laboratory (Bar Harbor, ME, USA), housed, and maintained in a 12:12 h light–dark cycle, with food and water available ad libitum. We chose C57BL/6 mouse for this study because it has been extensively used for electronic cigarette-induced inflammation and injury models in acute and chronic studies and reported to be susceptible to these exposures [30,31,32]. All the procedures in this protocol were approved by The Indiana University Bloomington Institutional Animal Care and Use Committee (IACUC) under protocol #22-013.

### 2.2. Electronic Cigarette Exposure

Adult male and female C57BL/6J mice were divided into four groups (n = 6/group; 3 males and 3 females), and treated for 14 days, as follows: group I: no treatment (cage control/room air control), group II: 0% nicotine in e-liquid (50% PG, 50% VG) [33], group III: 3% nicotine in e-liquid (50% PG, 50% VG), group IV: 6% nicotine in e-liquid (50% PG, 50% VG), using an inExpose™ full body chamber (SCIREQ, Montréal, QC, Canada). The inExpose™ system is a computerized inhalation machine that allows control of exposure doses [34,35]. The aerosols were generated by 1 puff/minute at a volume of 70 mL and a duration of 3.3 s with an e-cigarette (purchased from SCIREQ) setting at 230 °C and resistance of 1.5 ohm and pump flow rate 1 L/min. Exposures were conducted for 30 min/day. The humidity buffer chamber pulls room air at the rate of 1 L/min and dilutes the puff with the relative humidity in the air so that animals are not exposed to a completely dry aerosol. Additionally, as the flow is 2 L/min (including 1 L/min from the buffer chamber) the chambers have new air every 2.5 min. The bias flow ensures that the mice have a constant flow of air to prevent suffocation.

E-liquids for third and prior generation e-cigarettes contained ~1–4% (10–40 mg/mL) flavorings and 0.6–3% nicotine (6–30 mg/mL), though current fourth generation e-cigarettes can contain 5–7% (50–70 mg/mL) nicotine. Therefore, we wanted the average of doses which covers the concentration of nicotine in older to new generations of e-cigarettes [36,37,38]. All the e-liquids used in this study were purchased from an online vendor (Vapor Vapes, Sand City, CA, USA). Animals were euthanized on the day after the completion of the last exposure, and lung tissues were harvested and snap frozen in liquid nitrogen.

### 2.3. RNA Extraction

Frozen lung tissue was pulverized and homogenized. Total RNA was extracted with the Direct-zol kit (Zymo Research, Irvine, CA, USA). RNA quality and concentrations were checked with a NanoDrop spectrophotometer (Thermo Fisher Scientific, Waltham, MA, USA).

### 2.4. RT² Profiler PCR Array

A total of 100 ng of purified RNA were retrotranscribed with the RT^2^ First Strand Kit (Qiagen, Hilden, Germany). The expression of 84 NRs genes and coregulators (Table 1) was measured with the RT² Profiler^TM^ Array Mouse Receptors & Coregulators (PAMM-056YE) using SYBR green chemistry (RT² SYBR Green ROX qPCR Master mix, Qiagen) on a QuantStudio^®^ 5 Real-Time PCR System (Applied Biosystems, Waltham, MA, USA). Datasets were uploaded to the Gene Expression Omnibus (GEO) under accession GSE252208.

### 2.5. Data Analysis and Statistics

Data analysis was conducted using excel (2019, Microsoft 365) and RStudio 4.3.2, PBC, Boston, MA, USA. Fold changes were calculated with the 2^−ΔΔCT^ method [39], as described by us previously [40,41], using the average of a set of multiple endogenous control genes (Actb, B2m, Gapdh, Gusb, Hsp90ab1), as recommended by the manufacturer and prior publications [42]. Statistical significance was assessed using the Bioconductor limma package on R and the false discovery rate (FDR) was calculated with the Benjamini–Hochberg correction for multiple comparisons.

## 3. Results

### 3.1. Lung NR Expression in No Treatment (Control) vs. 0% Nicotine Exposed Males

**Figure 1:**
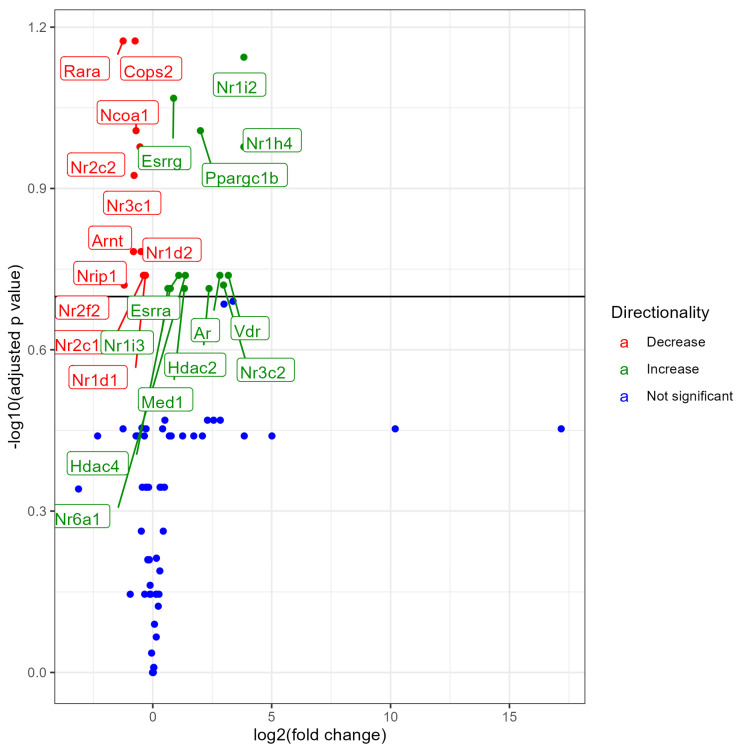
Volcano plot showing differential expression of NRs (both significant and non-significant) in male mice exposed to e-liquid with 0% nicotine vs. non-exposed controls. The plots are shown to visualize the most significantly expressed NRs. The plots were generated with the log2 fold change vs. the −log base 10 of the adjusted *p*-value (FDR). Labels indicate gene names, and colors indicate upregulation (green), downregulation (red), or no change (blue) compared to control. The horizontal line indicates the FDR < 0.20 threshold.

Male mice exposed to 0% nicotine e-liquid showed differential expression of 24 NRs, out of which 13 NRs showed upregulation and 11 NRs showed downregulation at an FDR < 0.2, when compared to control male mice (no treatment) (Figure 1). Upregulation of Ppargc1b, Nr1h4, Nr61a, Esrra, Ar, Vdr, Nr3c2, Hdac4, Nr1i3, Med1, and Hdac2, and downregulation of Rara, Cops2, Ncoa1, Nr2c2, Nr3c1, Nr1d2, Arnt, Nr2c1, Nr1d1, and Nr2f2, Nrip1 in male mice suggests that the expression of NRs is sensitive to e-liquid aerosol exposure. A list of all NRs’ expression levels along with the fold change values, *p* values, and FDR is provided in the Supplementary Materials (Table S1).

### 3.2. Lung NR Expression in No Treatment (Control) vs. 0% Nicotine Exposed Females

**Figure 2:**
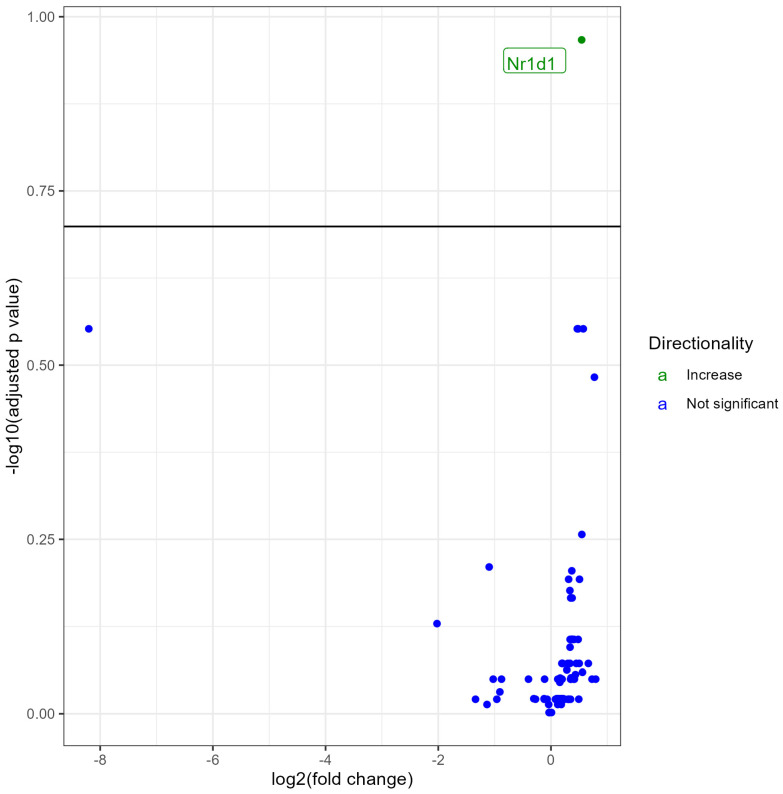
Volcano plot showing differential expression of NRs (both significant and non-significant) in female mice exposed to e-liquid with 0% nicotine vs. non-exposed controls. The plots are shown to visualize the most significantly expressed NRs. The plots were generated with the log2 fold change vs. the −log base 10 of the adjusted *p*-value (FDR). Labels indicate gene names, and colors indicate upregulation (green), or no change (blue) compared to control. The horizontal line indicates the FDR < 0.20 threshold.

Female mice exposed to 0% nicotine aerosols when compared with control female mice (no treatment) showed the significant upregulation of only 1 NR and no downregulated NRs at an FDR < 0.2 (Figure 2). We observed upregulation of Nr1d1. The fold change values, *p* values, and FDR values are provided in the Supplementary Materials (Table S2).

### 3.3. Lung NR Expression in No Nicotine (0% Nicotine) vs. 3% Nicotine Exposed Males

**Figure 3:**
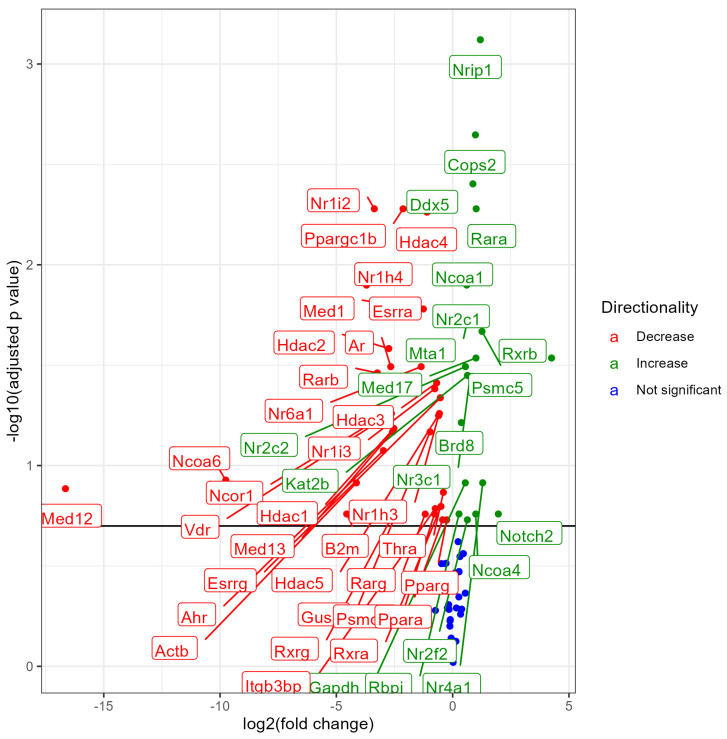
Volcano plot showing differential expression of NRs (both significant and non-significant) in male mice exposed to e-liquid with 0% nicotine vs. 3% nicotine. The plots are shown to visualize the most significantly expressed NRs. The plots were generated with the log2 fold change vs. the −log base 10 of the adjusted *p*-value (FDR). Labels indicate gene names, and colors indicate upregulation (green), downregulation (red), or no change (blue) compared to control. The horizontal line indicates the FDR < 0.20 threshold.

Male mice exposed to 3% nicotine e-liquid aerosols when compared with male mice exposed to 0% nicotine (no nicotine) e-liquid aerosols showed the differential expression of 53 NRs, out of which 20 NRs showed upregulation and 33 NRs showed downregulation at an FDR < 0.2 (Figure 3). The upregulation of Nrip1, Cops2, Ddx5, Rara, Ncoa1,Mta, Nr2c1, Psmc5, Rxrb, Med17, Nr2c2, Kat2b, Nr3c1, Brd8, Nr4a1, Gapdh, Notch2, Rbpj, Ncoa4, and Nr2f2, and downregulation of Nr1i2, ppargc1b, Hdac4, Nr1h4, Esrra, Med1, Hdac2, Nr6a1, Ar, Rarb, Hdac3, Nr1i3, Esrrg, Vdr, Hdac5,Ncor1, Nr1h3, hdac1, Med13, Gusb, AhR, Ncoa6, Actb, Med12, Thra, Rxrg, Rxra, Itgb3bp, Psmc3, Rarg, Pparg, and Ppara indicate a significant effect of nicotine in e-liquid. A list of all significantly modulated NRs along with the fold change, *p* values, and FDR is provided in the Supplement (Table S3).

### 3.4. Lung NR Expression in No Nicotine (0% Nicotine) vs. 3% Nicotine Exposed Females

**Figure 4:**
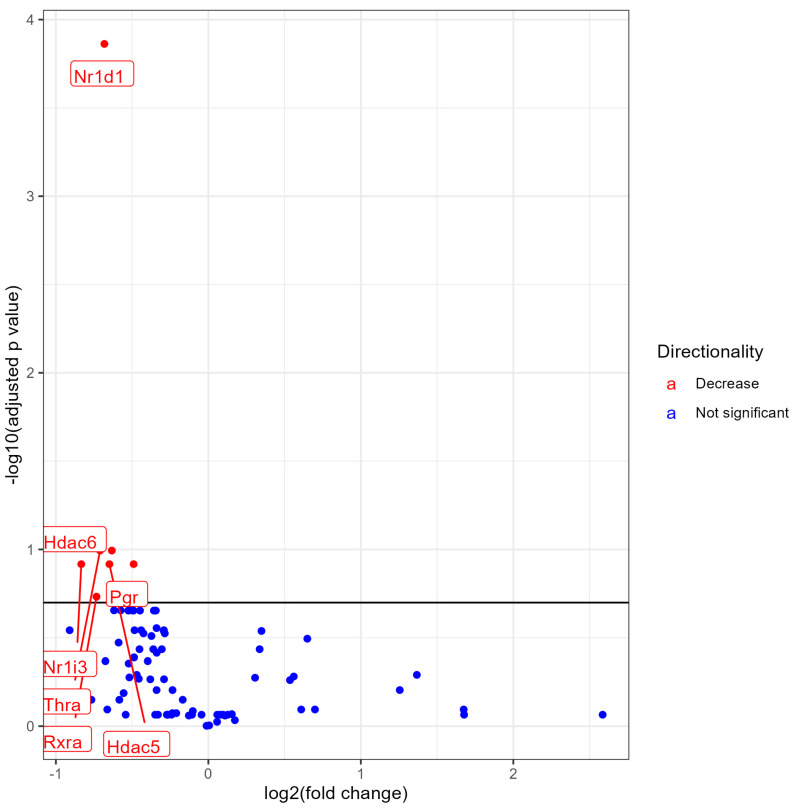
Volcano plot showing differential expression of NRs (both significant and non-significant) in female mice exposed to e-liquid with 0% nicotine vs. 3% nicotine. The plots are shown to visualize the most significantly expressed NRs. The plots were generated with the log2 fold change vs. the −log base 10 of the adjusted *p*-value. Labels indicate gene names, and colors indicate downregulation (red), or no change (blue) compared to control. The horizontal line indicates the FDR < 0.20 threshold.

Female mice exposed to 3% nicotine e-liquid aerosols when compared with female mice exposed to 0% nicotine (no nicotine) e-liquid aerosols showed the significant downregulation of seven NRs, namely, Nr1d1, Hra, Hdac6, Nr1i3, Pgr, Hdac5, and Rxra, and no upregulation at an FDR < 0.2 (Figure 4). A list of all significantly modulated NRs along with the fold change, *p* values, and FDR values is provided in the Supplementary Materials (Table S4).

### 3.5. Lung NR Expression No Nicotine (0% Nicotine) vs. 6% Nicotine Exposed Males

**Figure 5:**
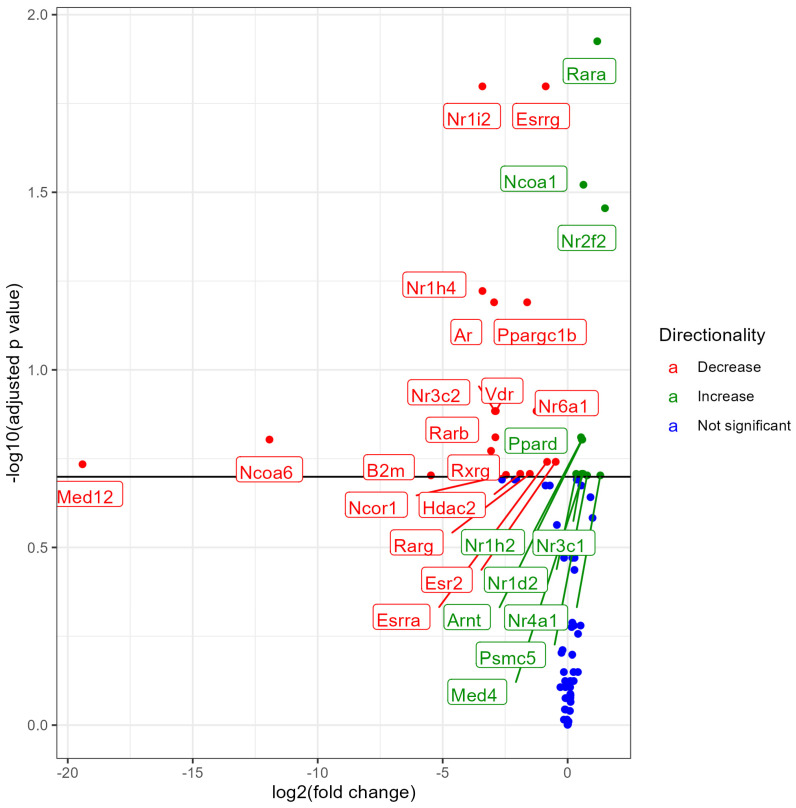
Volcano plot showing differential expression of NRs (both significant and non-significant) in male mice exposed to e-liquid with 0% nicotine vs. 6% nicotine. The plots are shown to visualize the most significantly expressed NRs. The plots were generated with the log2 fold change vs. the −log base 10 of the adjusted *p*-value (FDR). Labels indicate gene names, and colors indicate downregulation (red), upregulation (green), or no change (blue) compared to control. The horizontal line indicates the FDR < 0.20 threshold.

Male mice exposed to 6% nicotine e-liquid aerosols, when compared with male mice exposed to 0% nicotine (no nicotine) e-liquid aerosols, showed differential expression of 29Nrs out of which 11 NRs upregulated and 18 NRs downregulated at an FDR < 0.2. There was significant upregulation of Rara, Ncoa1, Nr2f2, Ppard, Nr1h2, Arnt, Nr3c1, Nr1d2, Med4, Nr4a1, and Psmc5, and significant downregulation of Nr1i2, Esrrg, Nr1h4, Ppargc1b, Ar, Nr3c2, Vdr, Nr6a1, Rarrb, Ncoa6, Rxrg, Esr2, Esrra, Med12, Hdac2, Rarg, Ncor1, and B2m (Figure 5). Results suggest the effect of nicotine on the expression of NRs. Interestingly, out of two nicotine groups (3% and 6%), the group exposed to a lesser concentration of nicotine (3%) showed the higher number of differentially expressed NRs in male mice (Figure 5). A list of all significantly modulated NRs along with the fold change, *p* values, and FDR values is provided in the Supplementary Materials (Table S5).

### 3.6. Lung NR Expression No Nicotine (0% Nicotine) vs. 6% Nicotine Exposed Females

**Figure 6:**
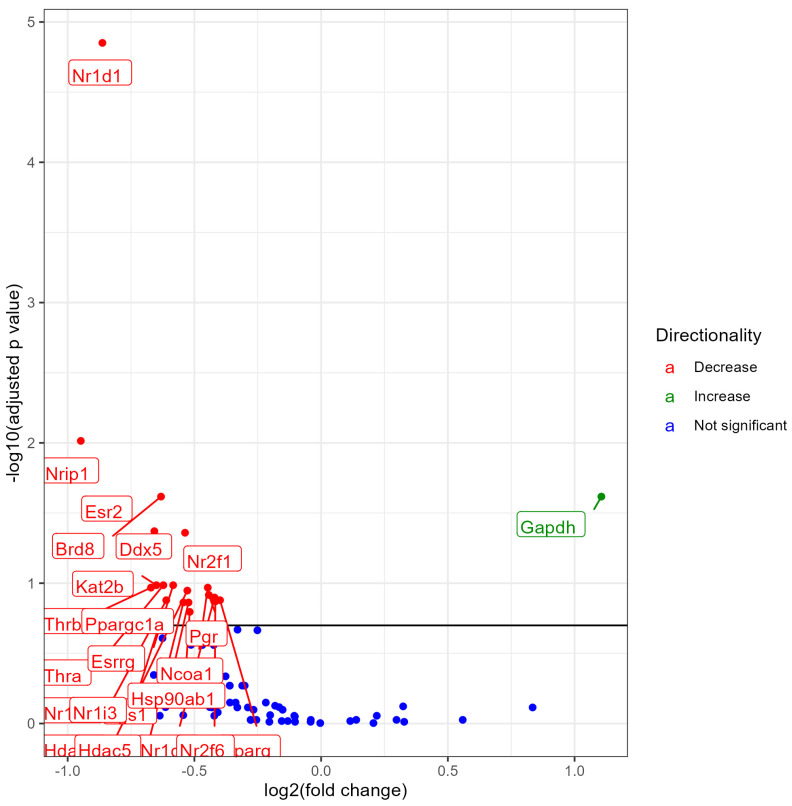
Volcano plot showing differential expression of NRs (both significant and non-significant) in female mice exposed to e-liquid with 0% nicotine vs. 3% nicotine. The plots are shown to visualize the most significantly expressed NRs. The plots were generated with the log2 fold change vs. the −log base 10 of the adjusted *p*-value (FDR). Labels indicate gene names, and colors indicate downregulation (red), upregulation (green), or no change (blue) compared to control. The horizontal line indicates the FDR < 0.20 threshold.

Female mice exposed to 6% nicotine e-liquid aerosols, when compared with male mice exposed to 0% nicotine (no nicotine) e-liquid aerosols, showed differential expression of 23 NRs out of which 1 NR upregulated and 22 NRs downregulated at an FDR < 0.2. There was significant downregulation of Nr1d1, Nrip1, Brd8, Esr2, Ddx5, Nr2f1, Thra, Esrrg, Kat2b, Thrb, Ncoa1, Hdac6, Pgr, Nr1d2, Pparg, Nr1h3, Nrf6, Tgs1, Hdac5, Ppargc1a, Hsp90ab1, and Nr1i3, and upregulation of Gapdh. Out of two nicotine groups (3% and 6%), the group exposed to a higher concentration of nicotine (6%) showed the higher number of differentially expressed NRs in female mice (Figure 6). A list of all significantly modulated NRs along with the fold change, *p* values, and FDR is provided in the Supplementary Materials (Table S6).

## 4. Discussion

The widespread promotion of ENDS as perceived “safer” alternatives to traditional tobacco products has led to a notable shift, especially among teenagers and young adults [2,3]. Despite lower observed toxic levels of ECCs in e-cigarette aerosols compared to cigarette smoke, ENDS, with their intricate design involving plastics, glass, and e-liquids, have the potential to introduce various EDCs such as such as phthalates, heavy metals, and flame retardants, to consumers [5,7]. Recent findings suggest that electronic cigarettes with specific flavors can induce inflammation and disrupt repair processes in lung cells [5,22]. Our aim in this study was to determine the sensitivity of NRs to ENDS aerosols in the presence or absence of nicotine. Given their ability to accommodate ligands, NRs are prime targets of man-made compounds that may mimic or antagonize the action of endogenous ligands. Therefore, NRs occupy a prominent role in OECD and EPA guidelines for testing and assessment of chemicals of concern including EDCs [27,28,29,43]. Considering that the susceptibility to these effects may be influenced by the sex variable, it is important to emphasize the need for comprehensive research that assesses the safety profile of ENDS in males and females.

The results from the current study indicate that NRs exhibit a high sensitivity to ENDS aerosols. The differential expression of NRs is evident in response to this exposure, irrespective of the presence of nicotine in e-liquids. Importantly, the doses of nicotine used in this study are physiologically relevant, as prior research has determined that a nicotine dose of 6 mg/kg is equivalent to what an ENDS user consumes in a day [44]. Moreover, several studies have used this dose of nicotine to check the physiochemical effects of electronic cigarette and tobacco cigarette exposure in mice and found that the effects of e-liquid aerosols containing 6–60 mg/kg/bw of nicotine are sex-specific [12,45]. Considering the sensitivity of NRs to low concentrations of toxicants, and to include a range of nicotine concentrations, we also included 3 mg/kg/bw of nicotine in the current study. Therefore, we used non-nicotine (0% nicotine) and nicotine (3% and 6%) ENDS aerosols, providing a range of nicotine exposure that is either equivalent or lesser than the daily human nicotine intentional exposure of an ENDS user.

Of the main differentially expressed NRs, AhR negatively regulates inflammation in A549 (human alveolar basal epithelial cells), modulating NF-κB signaling and controlling lung alveolar inflammation from airborne pollutants. Activated by benzo[a]pyrene, AhR influences gene expression and contributes to malignant cell transformation. Recent studies reveal its role in immune checkpoint regulation, urging cautious design of AhR ligands for safe and effective therapeutic use in inflammatory lung diseases [46,47,48,49,50]. This study reveals a downregulation of AhR in male mice exposed to a 3% nicotine e-liquid compared to those exposed to a 0% nicotine e-liquid. Øvrevik et al. found AhR and Arnt independently regulate chemokine responses to inhaled pollutants and infections. AhR inhibits p65 activation via a ligand-independent mechanism; Arnt may disrupt activated p65 [51]. In this study, the Arnt showed downregulation in the comparison of control and male mice exposed to e-liquids with 0% nicotine and upregulation in the comparison between male mice exposed to e-liquids with 0% vs. 6% nicotine.

In this investigation, AR also showed downregulation in the 3% and 6% nicotine exposed groups in males, when compared with 0% nicotine, while there was also an upregulation in males when comparing the control and 0% nicotine groups. This suggests a dose-dependent modification potentially mediated by NRs in male mice. Kalidhindi et al. [52] reported AR activation when treating gonadectomized mice with 5α-DHT, which also reduced Th2/Th17 inflammation and improved lung function in a model of mixed allergen (MA) challenge. Other studies have also reported AR contributions to lung inflammation using animal models [53,54].

Nr1i2 expression is noted in non-small cell lung cancer (NSCLC), providing a potential for targeted intervention using specific antagonists [55]. In our study, we observed downregulation of Nr1i2 in male mice, showing a decrease in 3% and 6% nicotine comparisons with 0% nicotine, while no significant difference was found in the control vs. 0% comparison.

Regarding Esrrg, we found a dose-dependent downregulation in males in the 0% vs. 3% and 0% vs. 6% nicotine comparisons, as well as in females. A study in old mice has shown that this NR modulates regulatory T cells through mitochondrial metabolism in the lungs [56]. We also observed a noteworthy increase in Nr4a1 expression in male mice in the 0% vs. 3% and 0% vs. 6% nicotine comparisons, while no differences were observed between the 0% and control groups, indicating a potential dose-dependent modification. Zhu et al. previously reported Nr4a1 as a novel regulator of acute lung injury induced by lipopolysaccharide (LPS), influencing mitochondrial fusion and necroptosis modulation [57]. Our data presented here also indicate that Nr1d1, a key circadian clock inhibitor, exhibits upregulation in females in both control vs. 0% and 0% vs. 3% nicotine, but downregulation in the 0–6% comparison. Notably, mice with null Nr5a1 genes exhibit the absence of adrenal glands and gonads, with male mice displaying female internal genitalia attributed to the loss of the Anti-Müllerian Hormone (AMH) during male sex differentiation [58]. Additionally, expression of Nr1d1 has been observed in NSCLC (non-small cell lung cancer) and presents an opportunity for targeted intervention using suitable antagonists in lung cancer cells. Kim et al. demonstrated that within the tumor microenvironment, Nr1d1 acts as a tumor suppressor by inhibiting the Nlrp3 inflammasome, indicating that activation of Nr1d1 to block the Nlrp3 inflammasome could potentially serve as a therapeutic approach against lung cancer [59]. Finally, the absence of the B2m gene correlates with immune escape by tumors and resistance to immunotherapy [60], and here we have found downregulation in male mice in a comparison of 0 and 6% nicotine exposure. In summary, NRs showed sensitivity towards the e-liquid aerosols regardless of the presence or absence of nicotine. However, when nicotine groups (3% and 6%) were compared with the non-nicotine e-liquid group (0%), expression patterns varied in males and females. For example, the total number of NRs expressed was higher in the 3% nicotine group in males, whereas the total number of NRs expressed was higher in the 6% nicotine group in females. The results from our pilot study indicate that expression patterns of NRs in all three groups (0%, 3%, 6% nicotine) exhibit both even and monotonous (females) and uneven and non-monotonous (males) dose responses. Such uneven dose responses are considered non-ideal and non-monotonous in males. These type of responses are consistent with the reported behavior of ECCs including EDCs [61,62], emphasizing the need to consider their presence in the evaluation of electronic cigarette effects.

Our presented pilot study has several limitations. First, it is important to acknowledge that the inExpose full body chamber may cause deposition on the fur of the animals and may lead to exposure of a higher concentration of constituents than the targeted concentrations. However, this possibility of exposure to higher doses uniformly applies to all the animals of all the exposure groups in this study. Therefore, this limitation should not have a significant bearing on our ability to compare groups and sexes. Another important aspect to be considered for the variation in the results is the operating conditions of e-cigarettes such as heating. The heating conditions used in this study (250 °C) are considered a non-burning operating condition and hence considered safe. However, according to the non-Arrhenius kinetics, nicotine degradation may be more rapidly initiated at low temperatures. Therefore, the electronic cigarettes with low heating temperatures may not qualify as safe [63]. Another limitation of this study is that we did not have an air-only control. Instead, a no-treatment group of animals was used as the control for non-nicotine (0%) group comparisons. A third limitation relies in our interpretation of the statistical analysis. We corrected the *p* values using the FDR method and arbitrarily selected FDR < 0.2 as the threshold for differential expression to interpret the implications of our results. A more stringent approach using FDR < 0.1 or <0.05 could be also considered to address this limitation (data available in supplementary tables) and interpret the results from this pilot and future studies. In this context, we would like to highlight that in some of the comparisons, a few housekeeping genes displayed differential expression at this FDR (Gapdh in males 0% vs. 3% nicotine, and in females 0% vs. 6% nicotine; Actb in males 0% vs. 3% nicotine). Therefore, we do not recommend using these individual genes when performing normalization of expression to a single housekeeping in the context of nicotine in mouse lung tissue.

While the present study does not aim to highlight any category of compounds, we predict the possible presence of chemicals of concern in ENDS in general, including EDCs, based on the NR gene expression effects observed. The major focus of this study is to test the ability of NR-gene-expression-based assays to be used as a screening method for the risk and exposure assessment of harmful chemicals in electronic cigarettes. This pioneering pilot study marks the first exploration of lung NR sensitivity to electronic cigarette exposure, yielding pilot data for further studies. This is a pilot study with the small exposure group sizes and limited range of nicotine concentration. Therefore, our results are indicative of the possibilities and need to be replicated and confirmed with larger exposure groups and increasing the range of nicotine concentrations. However, the findings provide a robust foundation for future investigations, enabling targeted studies on specific NR mechanisms within the lungs. It is crucial to acknowledge that further research is essential to delve into specific NRs and their downstream mechanisms, particularly in understanding their roles in inducing lung toxicities.

## 5. Conclusions

The current study underscores the significant public health concern posed by ENDS, which contain harmful substances such as EDCs. The sensitivity of NR-mediated mechanisms to e-cigarette exposure, irrespective of nicotine presence, is a noteworthy finding. The diverse response of NR expression patterns across sexes and between non-nicotine and different nicotine concentrations highlights the complexity of these mechanisms and warrants further confirmation studies. The observed alterations in NR expression may contribute to the adverse effects associated with e-cigarette use, emphasizing the necessity for further research on NRs as potential biomarkers for risk and exposure assessment in the context of e-cigarettes. Furthermore, the results revealed sex differences in NR-mediated e-cigarette toxicities, with distinct responses in male and female mice. This sex-specific variability adds complexity to understanding the impact of e-cigarettes on the endocrine system. Our findings provide a rationale to further investigate role of sex into the potential risks linked to e-cigarette exposure and emphasize the need for comprehensive research to assess the safety profile of electronic nicotine delivery systems. The study suggests that e-cigarette exposure may induce NR-mediated lung toxicities, prompting further investigation into the use of NRs as biomarkers for risk and exposure assessment. The sensitivity of these mechanisms to various chemicals, including EDCs, makes it particularly intriguing for future research aimed at enhancing our understanding of the potential health risks associated with electronic cigarette use.

## Figures and Tables

**Table 1:** List of RT^2^ Profiler PCR array ‘s 84 NRs and coregulators.

Symbol	Description
AhR	aryl-hydrocarbon receptor
Ar	androgen receptor
Arnt	Aryl-hydrocarbon receptor nuclear translocator
Brd8	bromodomain containing 8
Cops2	COP9 (constitutive photomorphogenic) homolog, subunit 2
Crebbp	CREB binding protein
Ddx5	DEAD (Asp-Glu-Ala-Asp) box polypeptide 5
Esr1	estrogen receptor 1 (alpha)
Esr2	estrogen receptor 2 (beta)
Esrra	estrogen-related receptor, alpha
Esrrb	estrogen-related receptor, beta
Esrrg	estrogen-related receptor gamma
Hdac1	histone deacetylase 1
Hdac2	histone deacetylase 2
Hdac3	histone deacetylase 3
Hdac4	histone deacetylase 4
Hdac5	histone deacetylase 5
Hdac6	histone deacetylase 6
Hdac7	histone deacetylase 7
Hnf4a	hepatic nuclear factor 4, alpha
Itgb3bp	integrin beta 3 binding protein (beta3-endonexin)
Kat2b	K(lysine) acetyltransferase 2B
Kat5	K(lysine) acetyltransferase 5
Med1	mediator complex subunit 1
Med12	Mediator of RNA polymerase II transcription, subunit 12 homolog (yeast)
Med13	mediator complex subunit 13
Med14	mediator complex subunit 14
Med16	mediator complex subunit 16
Med17	mediator complex subunit 17
Med24	mediator complex subunit 24
Med4	Mediator of RNA polymerase II transcription, subunit 4 homolog (yeast)
Mta1	metastasis associated 1
Ncoa1	nuclear receptor coactivator 1
Ncoa2	nuclear receptor coactivator 2
Ncoa3	nuclear receptor coactivator 3
Ncoa4	nuclear receptor coactivator 4
Ncoa6	nuclear receptor coactivator 6
Ncor1	nuclear receptor co-repressor 1
Ncor2	nuclear receptor co-repressor 2
Nfkb2	nuclear factor of kappa light polypeptide gene enhancer in B cells 2, p49/p100
Nono	non-POU-domain-containing, octamer binding protein
Notch2	notch 2
Nr0b1	nuclear receptor subfamily 0, group B, member 1
Nr0b2	nuclear receptor subfamily 0, group B, member 2
Nr1d1	nuclear receptor subfamily 1, group D, member 1
Nr1d2	nuclear receptor subfamily 1, group D, member 2
Nr1h2	nuclear receptor subfamily 1, group H, member 2
Nr1h3	nuclear receptor subfamily 1, group H, member 3
Nr1h4	nuclear receptor subfamily 1, group H, member 4
Nr1i2	nuclear receptor subfamily 1, group I, member 2
Nr1i3	nuclear receptor subfamily 1, group I, member 3
Nr2c1	nuclear receptor subfamily 2, group C, member 1
Nr2c2	nuclear receptor subfamily 2, group C, member 2
Nr2e3	nuclear receptor subfamily 2, group E, member 3
Nr2f1	nuclear receptor subfamily 2, group F, member 1
Nr2f2	nuclear receptor subfamily 2, group F, member 2
Nr2f6	nuclear receptor subfamily 2, group F, member 6
Nr3c1	nuclear receptor subfamily 3, group C, member 1
Nr3c2	nuclear receptor subfamily 3, group C, member 2
Nr4a1	nuclear receptor subfamily 4, group A, member 1
Nr5a1	nuclear receptor subfamily 5, group A, member 1
Nr6a1	nuclear receptor subfamily 6, group A, member 1
Nrip1	nuclear receptor interacting protein 1
Pgr	progesterone receptor
Ppara	peroxisome proliferator activated receptor alpha
Ppard	peroxisome proliferator activator receptor delta
Pparg	peroxisome proliferator activated receptor gamma
Ppargc1a	peroxisome proliferative activated receptor, gamma, coactivator 1 alpha
Ppargc1b	peroxisome proliferative activated receptor, gamma, coactivator 1 beta
Psmc3	proteasome (prosome, macropain) 26S subunit, ATPase 3
Psmc5	protease (prosome, macropain) 26S subunit, ATPase 5
Rara	retinoic acid receptor, alpha
Rarb	retinoic acid receptor, beta
Rarg	retinoic acid receptor, gamma
Rbpj	recombination signal binding protein for immunoglobulin kappa J region
Rora	RAR-related orphan receptor alpha
Rxra	retinoid X receptor alpha
Rxrb	retinoid X receptor beta
Rxrg	retinoid X receptor gamma
Tgs1	trimethylguanosine synthase homolog (S. cerevisiae)
Thra	thyroid hormone receptor alpha
Thrb	thyroid hormone receptor beta
Trip4	thyroid hormone receptor interactor 4
Vdr	vitamin D receptor
Actb	actin, beta
B2m	beta-2 microglobulin
Gapdh	glyceraldehyde-3-phosphate dehydrogenase
Gusb	glucuronidase, beta
Hsp90ab1	Heat shock protein 90 alpha (cytosolic), class B member 1

## Data Availability

Data and code can be retrieved here: https://github.com/psilveyra/vapingnicotine.git (accessed on 10 May 2024).

## Supplementary Materials

The following supporting information can be downloaded at: https://www.mdpi.com/article/10.3390/ijerph21060810/s1, Table S1: Number of significant fold changes, no treatment control vs. 0% nicotine (Male), Table S2: Number of significant fold changes, no treatment control vs. 0% nicotine (female), Table S3: Number of significant fold changes, 0% nicotine vs. 3% nicotine (Male), Table S4: Number of significant fold changes, 0% nicotine vs. 3% nicotine (female), Table S5: Number of significant fold changes, 0% nicotine vs. 6% nicotine (Male), Table S6: Number of significant fold changes, 0% nicotine vs. 6% nicotine (female).
