# Supplementary material for: Sensitivity of Mouse Lung Nuclear Receptors to Electronic Cigarette Aerosols and Influence of Sex Differences: A Pilot Study
Source: Int J Environ Res Public Health. 2024 Jun 20;21(6):810. doi: 10.3390/ijerph21060810 (PMC11203813; doi:10.3390/ijerph21060810)
Supplement: Supplementary file 1 [file ijerph-21-00810-s001.zip › ijerph-3028246-supplementary.pdf]

Table S1: Number of significant fold changes, no treatment control vs 0% nicotine (Male)

Target	Adjusted p.value	Log fold change
Rara	0.067	-1247
Cops2	0.067	-0.743
Nr1i2	0.072	3.831
Esrrg	0.086	0.877
Ncoa1	0.098	-0.703
Ppargc1b	0.098	2.005
Nr2c2	0.105	-0.539
Nr1h4	0.105	3.830
Nr3c1	0.119	-0.786
Nr1d2	0.165	-0.492
Arnt	0.165	-0.811
Nr2c1	0.183	-0.360
Nr6a1	0.183	1.371
Esrra	0.183	1.092
Nr1d1	0.183	-0.307
Ar	0.183	2.823
Nrip1	0.183	-0.39
Vdr	0.183	3.179
Nr3c2	0.190	2.975
Nr2f2	0.190	-1.203
Hdac4	0.193	0.738
Nr1i3	0.193	0.636
Med1	0.193	1.331
Hdac2	0.193	2.366

Table S2: Number of significant fold changes, no treatment control vs 0% nicotine (female)

Target	Adjusted p.value	Log fold change
Nr1d1	0.108	0.545

Table S3: Number of significant fold changes, 0% nicotine vs 3% nicotine (Male)

Target	Adjusted p.value	Log fold change
Nrip1	0.001	1.190
Cops2	0.002	0.978
Ddx5	0.004	0.870
Nr1i2	0.005	-3.368
Rara	0.005	1.011
Ppargc1b	0.005	-2.131
Hdac4	0.005	-1.103
Ncoa1	0.013	0.603

Nr1h4	0.013	0.603
Esrra	0.017	-1.260
Med1	0.017	-1.652
Mta1	0.017	0.749
Nr2c1	0.017	0.430
Psmc5	0.021	1.261
Hdac2	0.026	-2.747
Rxrb	0.029	4.251
Med17	0.029	1.001
Nr6a1	0.029	1.001
Nr2c2	0.032	0.549
Ar	0.032	-2.662
Rarb	0.035	-3.239
Kat2b	0.036	0.637
Nr3c1	0.036	0.749
Hdac3	0.039	-0.687
Nr1i3	0.041	-0.769
Esrrg	0.046	-0.530
Vdr	0.055	-2.637
Hdac5	0.055	-0.570
Ncor1	0.056	-2.955
Nr1h3	0.056	-0.604
Brd8	0.061	0.372
Hdac1	0.065	-2.526
Med13	0.068	-2.587
Gusb	0.068	-0.973
Ahr	0.084	-2.980
Ncoa6	0.118	-9.755
Nr4a1	0.122	1.292
Gapdh	0.122	0.543
Actb	0.122	-4.139
Med12	0.130	-16.647
Thra	0.136	-0.399
Rxrg	0.156	-2.331
Rxra	0.160	-0.503
Itgb3bp	0.164	-0.749
Notch2	0.174	1.964
Rbpj	0.174	0.273
B2m	0.174	1.964
Ncoa4	0.174	0.991
Psmc3	0.174	-0.783
Rarg	0.174	-1.180
Pparg	0.186	-0.432
Ppara	0.186	-0.432
Nr2f2	0.186	0.632

Table S4: Number of significant fold changes, 0% nicotine vs 3% nicotine (female)

Target	Adjusted p.value	Log fold change
Nr1d1	0.000	-0.682
Thra	0.101	-0.710
Hdac6	0.101	-0.633
Nr1i3	0.121	-0.834
Pgr	0.121	-0.490
Hdac5	0.121	-0.649
Rxra	0.185	-0.734

Table S5: Number of significant fold changes, 0% nicotine vs 6% nicotine (Male)

Target	Adjusted p.value	Log fold change
Rara	0.012	1.185
Nr1i2	0.016	-3.411
Esrrg	0.016	0.878
Ncoa1	0.030	0.631
Nr2f2	0.035	1.493
Nr1h4	0.060	-3.409
Ppargc1b	0.065	-1.620
Ar	0.065	-2.943
Nr3c2	0.131	-2.908
Vdr	0.131	-2.875
Nr6a1	0.131	-1.245
Ppard	0.155	0.535
Rarb	0.155	-2.891
Ncoa6	0.157	-11.930
Nr1h2	0.157	0.567
Arnt	0.157	0.586
Rxrg	0.169	-3.066
Esr2	0.182	-0.478
Esrra	0.182	-0.823
Med12	0.184	-19.409
Hdac2	0.196	-1.898
Rarg	0.196	-1.898
Nr3c1	0.196	-1.898
Nr1d2	0.196	0.562
Med4	0.196	0.617
Ncor1	0.198	-2.467
Nr4a1	0.198	1.307
Psmc5	0.198	0.779
B2m	0.198	-5.470

Table S6: Number of significant fold changes, 0% nicotine vs 6% nicotine (female)

Target	Adjusted p.value	Log fold change
Nr1d1	0.000	-0.863
Nrip1	0.010	-0.948
Brd8	0.024	-0.632
Gapdh	0.024	1.106
Esr2	0.027	-0.812
Ddx5	0.043	-0.658
Nr2f1	0.044	-0.537
Thra	0.103	-0.622
Esrrg	0.103	-0.584
Kat2b	0.103	-0.650
Thrb	0.108	-0.671
Ncoa1	0.108	-0.447
Hdac6	0.113	-0.528
Pgr	0.121	-0.443
Nr1d2	0.127	-0.420
Pparg	0.132	-0.399
Nr1h3	0.132	-0.399
Nr2f6	0.135	-0.417
Tgs1	0.137	-0.523
Hdac5	0.137	-0.543
Ppargc1a	0.160	-0.784
Hsp90ab1	0.160	-0.518
Nr1i3	0.172	-0.627
